# Sleep Abnormalities Among Patients With and Without Diabetes Using Pittsburg Sleep Quality Index and Epworth Sleepiness Scale

**DOI:** 10.7759/cureus.2151

**Published:** 2018-02-04

**Authors:** Ujala Zubair, Fatima Majid, Adeel A Siddiqui, Zarafshan Zubair

**Affiliations:** 1 Medicine, Dow University of Health Sciences (DUHS), Karachi, Pakistan; 2 Student at Kmdc, Abbasi shaheed hospital; 3 Orthopedic Surgery, Dow University of Health Sciences (DUHS), Karachi, Pakistan; 4 MBBS, Dow University of Health Sciences (DUHS), Karachi, Pakistan

**Keywords:** sleep abnormalities, type 2 diabetes mellitus, pittsburg sleep quality index, epworth sleepiness scale

## Abstract

Introduction

Diabetes has a great influence on sleep patterns. Several hormonal mechanisms are disrupted in patients with diabetes and, hence, affect their sleep patterns. Sleep disturbances further worsen the state of the disease itself.

Method

In this cross-sectional study, we collected data from 50 healthy adults and 50 patients diagnosed with type 2 diabetes mellitus without comorbidities. Study participants were asked to complete the Pittsburgh Sleep Quality Index (PSQI) and Epworth Sleepiness Scale (ESS) surveys.

Results

The mean PSQI score was 8.64 ± 3.96 for patients with type 2 diabetes and 4.24 ± 2.72 for patients without diabetes. The mean Epworth Sleepiness score was 6.3 ± 5.29 among patients with diabetes and 1.94 ± 2.34 for patients without diabetes.

Conclusion

The early diagnosis and management of sleep problems can help maintain target blood glucose levels and may help impede the future development of complications.

## Introduction

Sleep is a bio-behavioral phenomenon that regulates many important mechanisms within the human body. Sleep regulates the hormones involved in regulating blood glucose levels. Glucose tolerance and insulin sensitivity are at their peak in the morning. As the day passes, blood glucose levels increase and insulin sensitivity decreases to its lowest level, which occurs overnight. During sleep deprivation, alterations in glucose tolerance and insulin sensitivity occur from morning to evening in people with type 2 diabetes mellitus as well as obese people without type 2 diabetes due to a disturbance of the circadian changes in cortisol causing reduced beta cell sensitivity. Beta cells then release insulin continuously, causing insulinopenia later in life, which can predispose a patient to develop diabetes [1].

The most restorative sleep stage is non-rapid eye movement sleep or slow-wave sleep (SWS). During SWS, brain glucose consumption decreases, growth hormone is released, and corticotropic hormones are inhibited. All these phenomena increase insulin sensitivity. Tasali et al. showed that the suppression of SWS results in decreased insulin sensitivity, resulting in reduced glucose tolerance [2].

Inadequate sleep increases the levels of ghrelin and leptin in the blood, which work to increase appetite and reduce energy expenditure causing obesity. Leptin levels were increased in obstructive sleep apnea patients by about 50% as compared to healthy individuals [1,3-4]. Short sleep activates low-grade inflammation. In a recent study, 30 healthy individuals were allowed to sleep for only four hours. They had increased levels of IL-6 and tumor necrosis factor-alpha as a result. These two mediators are associated with insulin resistance. Many other inflammatory cytokines are also involved, increasing the risk for cardiovascular disease in patients with diabetes [5].

Neuropathic pain is worse at night and is responsible for a significant reduction in the amount of SWS a patient receives. Among people with neuropathy, 24.9% to 50% had poor sleep quality [6-7]. The prevalence of obstructive sleep apnea is 36% in patients with type 2 diabetes. This ratio is more than double the prevalence of obstructive sleep apnea in healthy individuals [8].

## Materials and methods

We conducted this cross-sectional study in Karachi from November 2016 to January 2017. We collected data from 50 healthy adults and 50 patients diagnosed with type 2 diabetes mellitus without comorbidities. All study participants were instructed to complete the Pittsburgh Sleep Quality Index (PSQI) and Epworth Sleepiness Scale (ESS) surveys. The PSQI is a self-rated questionnaire assessing seven components of sleep, covering subjective sleep quality, sleep latency, sleep duration, habitual sleep efficiency, sleep disturbances, use of sleeping medications, and daytime dysfunction. The ESS assessment consists of eight questions regarding daytime sleepiness, including the chances of dozing during sitting, reading, talking, being a passenger, sitting quietly, and at times in the afternoon.

## Results

Data were collected from 100 individuals (50 healthy with no comorbidities, 50 with type 2 diabetes mellitus). Participants with coexisting hypertension or another disorder were excluded from the study. The mean age of study participants was 50 ± 7 years (range, 26 to 70 years). The mean age of patients with diabetes was 51 ± 7 years; the mean age of healthy participants was 49 ± 7 years. Twenty men and 27 women were in the diabetes group, and 18 men and 32 women were in the healthy group.

**Figure 1:**
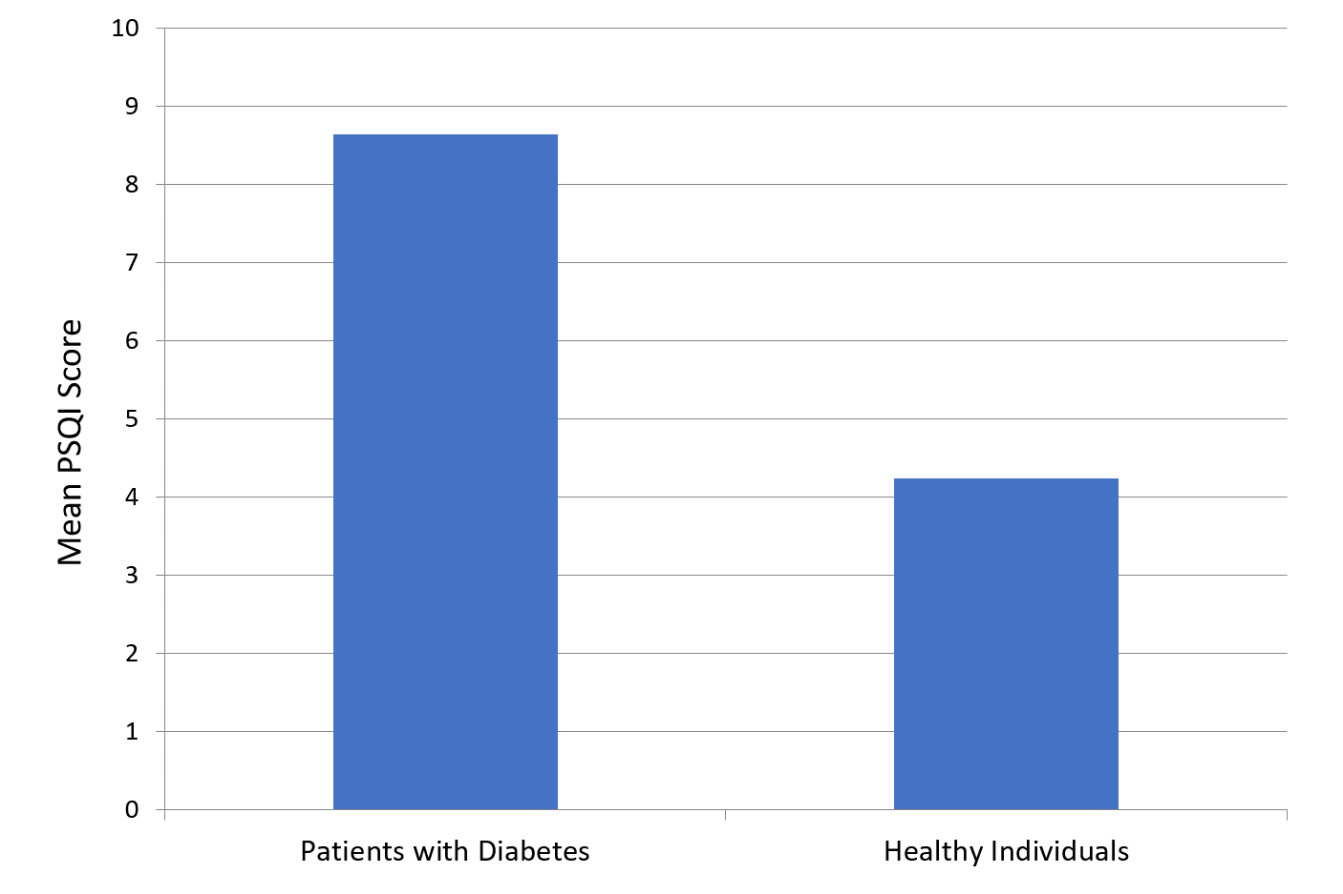
Comparison of global Pittsburgh Sleep Quality Index score among patients with and without diabetes 0 - 4 = no difficulty with sleep >5 = difficulty in sleep Abbreviation: PSQI, Pittsburgh Sleep Quality Index

**Figure 2:**
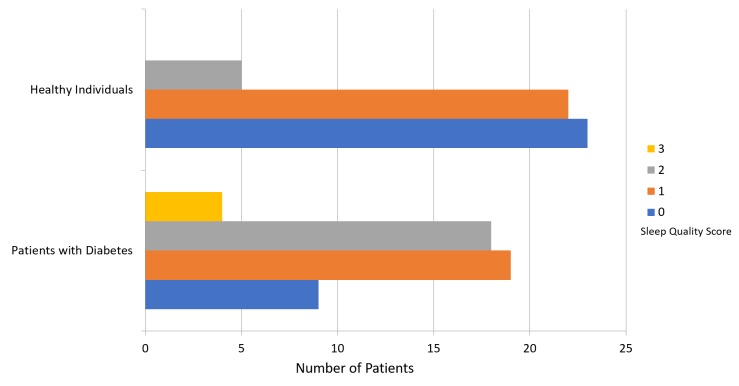
Subjective sleep quality in patients with and without diabetes 0 = very good 1 = fairly good 2 = fairly bad 3 = very bad

**Figure 3:**
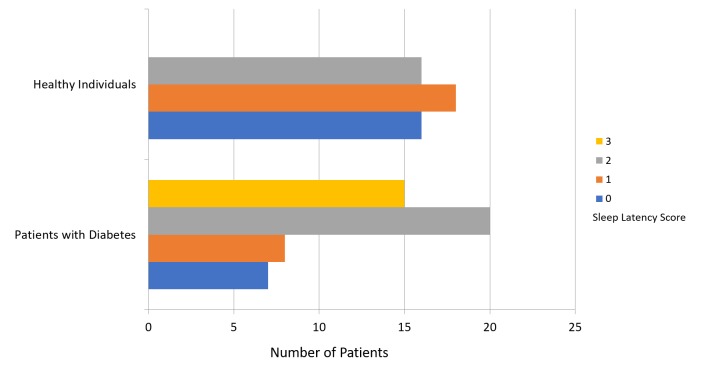
Sleep latency scores in patients with and without diabetes 0 = score 0 out of 6 1 = score 1-2 2 = score 3-4 3 = score 5-6

**Figure 4:**
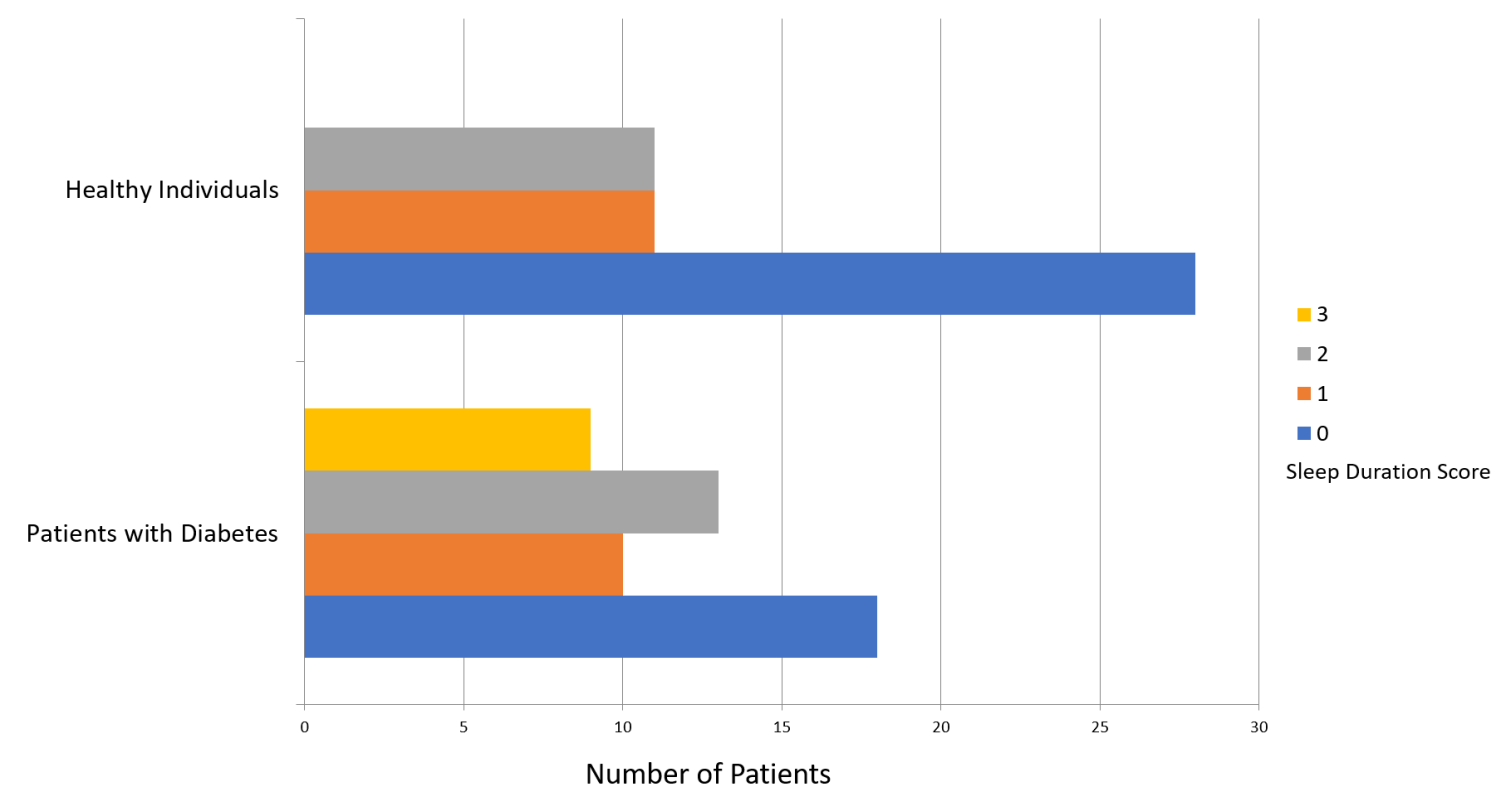
Sleep duration scores in patients with and without diabetes 0 = > 7 hours 1 = 6-7 hours 2 = 5-6 hours 3 = <5 hours

**Figure 5:**
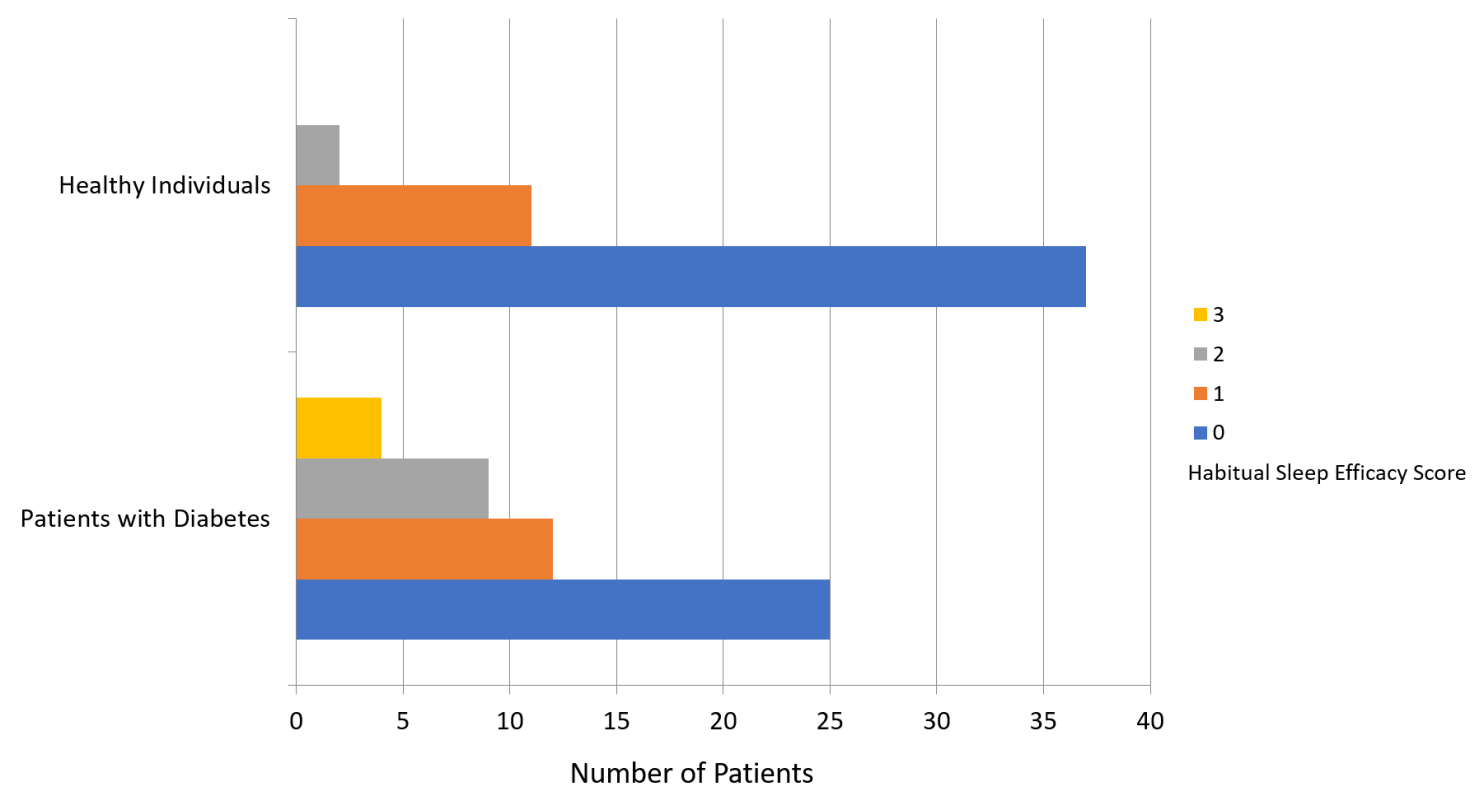
Habitual sleep efficacy scores in patients with and without diabetes 0 = >85% 1 = 75-84% 2 = 65-74% 3 = <65%

**Figure 6:**
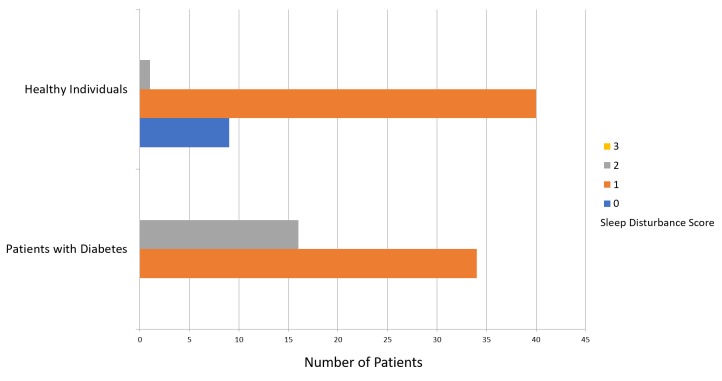
Sleep disturbances in patients with and without diabetes 0 = score 0 out of 27 1 = score 1-9 2 = score 10-18 3 = score of 19-27

**Figure 7:**
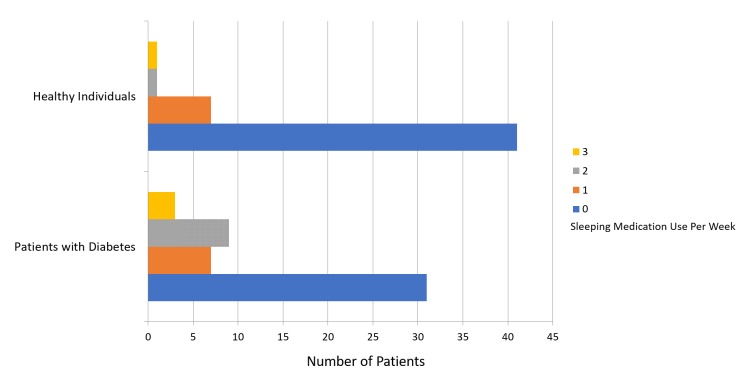
Use of sleeping medications in patients with and without diabetes 0 = not during the past month 1 = less than once a week 2 = once or twice a week 3 = three or more times a week

**Figure 8:**
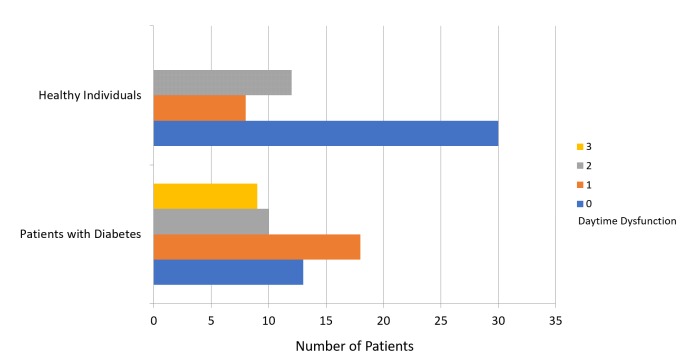
Daytime dysfunction in patients with and without diabetes 0 = score of 0 out of 6 1 = score 1-2 2 = score 3-4 3 = score 5-6

**Figure 9:**
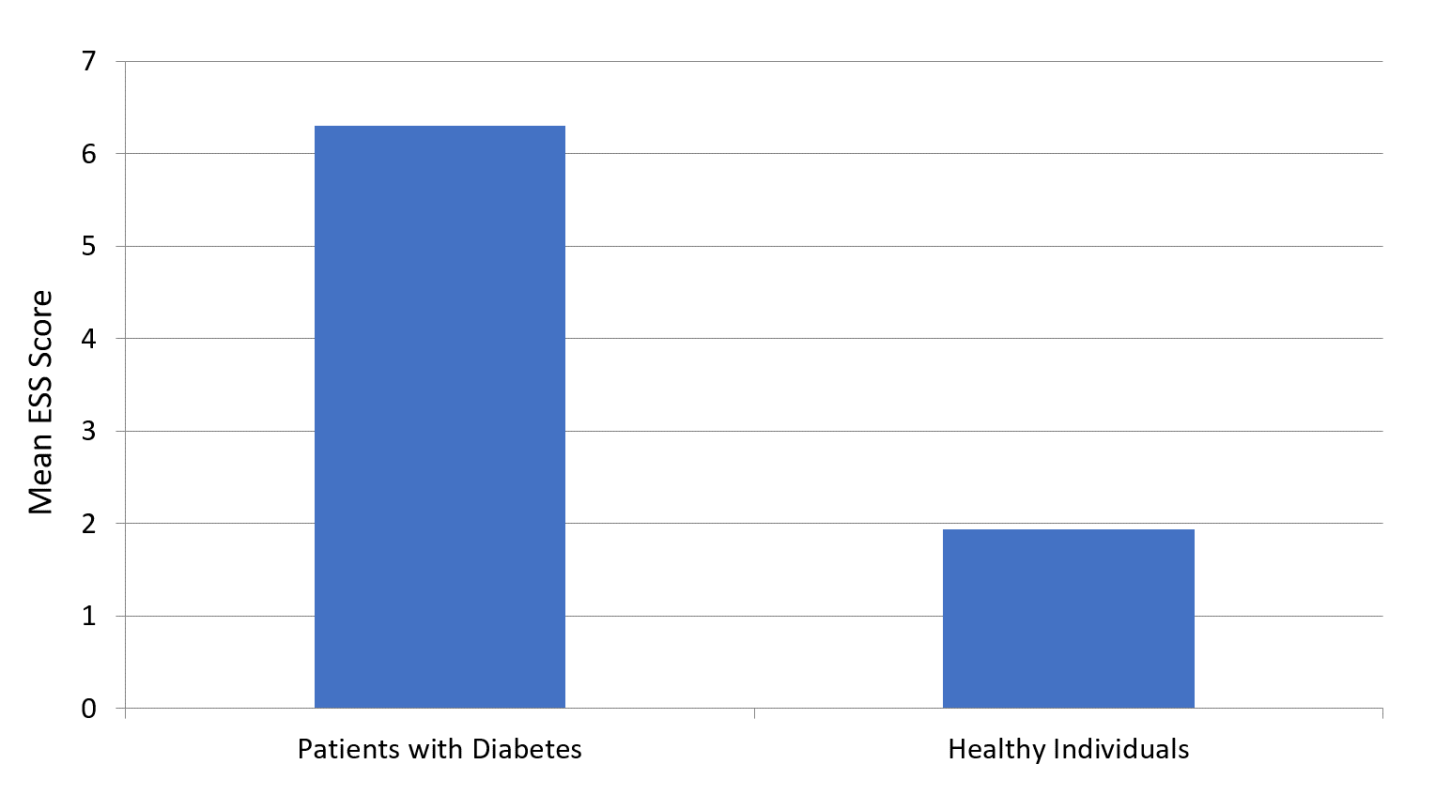
Epworth Sleepiness Scale mean scores in patients with and without diabetes score 1-6 = Individual is getting enough sleep 7-8 = Individual is getting average sleep >9 = Medical help is required Abbreviation: ESS, Epworth Sleepiness Scale

The mean PSQI score was 8.64 ± 3.96 for patients with diabetes and 4.24 ± 2.72 for participants in the healthy group (Figure 1). Figure 2 presents a comparison of subjective sleep quality; Figure 3 presents a comparison of sleep latency; Figure 4 presents a comparison of sleep duration; Figure 5 presents a comparison of habitual sleep efficacy; Figure 6 presents a comparison of sleep disturbance; Figure 7 presents a comparison of sleeping medication use; and Figure 8 presents a comparison of daytime dysfunction between patients with and without diabetes. The mean ESS score was 6.3 ± 5.29 for patients with diabetes and 1.94 ± 2.34 for participants in the healthy group (Figure 9).

## Discussion

In one study, participant sleep was restricted from 10 to four hours, and glucose infusions were administered in the morning. Insulin levels for patients getting four hours of sleep were low compared to patients getting 10 hours of sleep [9]. Another study showed a marked reduction in insulin sensitivity and glucose tolerance after eight nights of five-hour sleep sessions compared to the insulin sensitivity and glucose tolerance associated with eight-hour sleep sessions (unpublished poster; Leproult R, Holmback U, Van Cauter E: Marked decrease in insulin sensitivity following one week of partial sleep deprivation with or without circadian misalignment. First Annual Chicago Diabetes Day, June 17, 2006. University of Chicago, Chicago, USA). We found a mean PSQI score of 8.64 among patients with diabetes (standard deviation (SD), 3.96). Lopes et al. reported a mean PSQI score of 6.29 ± 3.84 [6]. Poor sleep quality (a PSQI score > 6) was present in 45% of the patients and was associated with age, restless leg syndrome, and peripheral neuropathy [6]. In a Korean study of 784 patients with type 2 diabetes, 38.4% of patients had PSQI scores > 5, indicating poor sleep quality [10]. In an Indian study of 120 patients with type 2 diabetes, the mean PSQI score was 7.08 (SD, 3.89), and 69% of patients had PSQI > 5 [11]. Another study reported poor sleep quality among 48% of patients with type 2 diabetes, of which the poorest sleep quality occurred in patients who had been diagnosed with diabetes for over 10 years [12].

In our study, 24% patients with diabetes reported using sleeping medications at least once weekly whereas only 4% healthy patients reported using sleeping medications at least once weekly. The frequent use of sleeping medications could worsen the sleep-wake cycle and lead to stress [13].

Our study was limited by not recording the duration of diabetes diagnoses for the participants. The duration of diabetes has a vast influence on sleep quality. Our study was also limited by a relatively small sample size.

## Conclusions

Sleep problems related to diabetes are associated with a poor quality of life. Diagnosing and managing sleep problems early can help maintain blood glucose levels within targeted ranges and may impede the development of complications.
